# Human pancreatic adenocarcinoma contains a side population resistant to gemcitabine

**DOI:** 10.1186/1471-2407-12-354

**Published:** 2012-08-15

**Authors:** Anke Van den broeck, Lies Gremeaux, Baki Topal, Hugo Vankelecom

**Affiliations:** 1 Department of Abdominal Surgery, University Hospitals Leuven, Herestraat 49, B-3000 Leuven, Belgium; 2 Laboratory of Tissue Plasticity, Research Unit of Embryo and Stem Cells, Department of Development & Regeneration, University of Leuven (KU Leuven), Leuven, Belgium

**Keywords:** Pancreatic cancer, Chemoresistance, Side population

## Abstract

**Background:**

Therapy resistance remains one of the major challenges to improve the prognosis of patients with pancreatic cancer. Chemoresistant cells, which potentially also display cancer stem cell (CSC) characteristics, can be isolated using the side population (SP) technique. Our aim was to search for a SP in human pancreatic ductal adenocarcinoma (PDAC) and to examine its chemoresistance and CSC(−like) phenotype.

**Methods:**

Human PDAC samples were expanded in immunodeficient mice and first-generation xenografts analyzed for the presence of a Hoechst dye-effluxing SP using flow cytometry (FACS). To investigate chemoresistance of the SP, mice bearing PDAC xenografts were treated with gemcitabine and SP proportion determined. In addition, the SP and the main tumour cell population (MP) were sorted by FACS for RNA extraction to profile gene expression, and for culturing under sphere-forming conditions.

**Results:**

A SP was identified in all PDAC samples, analyzed. This SP was more resistant to gemcitabine than the other tumour cells as examined *in vivo*. Whole-genome expression profiling of the SP revealed upregulation of genes related to therapy resistance, apoptotic regulation and epithelial-mesenchymal transition. In addition, the SP displayed higher tumourigenic (CSC) activity than the MP as analyzed *in vitro* by sphere-forming capacity.

**Conclusion:**

We identified a SP in human PDAC and uncovered a chemoresistant and CSC-associated phenotype. This SP may represent a new therapeutic target in pancreatic cancer.

**Trial registration:**

Clinicaltrials.gov NCT00936104

## Background

Pancreatic cancer or ductal adenocarcinoma (PDAC) remains a highly lethal disease. Despite improvements in medical and surgical care, the overall 5-year survival still has not exceeded 5% [1]. Resistance to chemotherapy is a major cause of treatment failure in pancreatic cancer, both in adjuvant setting after intended curative surgery as well as in advanced inoperable stages [2]. Therefore, PDAC’s chemoresistant cells are highly wanted targets for new therapeutic strategies to eventually improve overall survival.

Therapeutic resistance in pancreatic cancer is caused by low permeability of the tumour micro-environment, as well as by the efficient efflux of toxic agents [3,4]. One approach to isolate drug-effluxing cells is provided by the side population (SP) technique [5]. SP cells are identified on the basis of Hoechst-dye efflux capacity because of the presence of multidrug resistance transporters. Cells that expel the dye are visualized by dual-wavelength flow cytometry (FACS) as a ‘Hoechst low’ tail of cells, the SP, relative to a larger bulk of ‘Hoechst high’ cells, the main population (MP).

Recently, a SP has been identified in cultured pancreatic cancer cell lines and was found to be chemoresistant to gemcitabine treatment as evaluated *in vitro* in these cultured cell lines [3,6-8]. To date, it is not known whether clinical human PDAC contains a SP and wether this SP is resistant to gemcitabine when assessed *in vivo*.

In multiple types of cancer the SP is enriched in cells displaying properties of cancer stem cells (CSC) [9,10]. By definition, CSC (also referred to as tumour-driving cells) represent the tumour’s subpopulation with the highest capacity to drive its growth, invasion and metastasis. CSC are also considered responsible for therapy resistance and disease recurrence [11], and may therefore represent interesting targets for new and more effective treatment strategies [12]. Although still subject of debate, (candidate) CSC populations are being identified in a growing number of cancer types and their functional significance has recently been strongly supported [13-15]. In pancreatic cancer, cells based on specific cell-surface markers (i.e. CD24^+^CD44^+^ESA^+^ and CD133^+^ cells) have been reported to possess CSC characteristics [12,16,17].

In the present study, we report for the first time that human PDAC contains a SP and analyzed its resistance to the current standard chemotherapeutic agent for pancreatic cancer, gemcitabine, using a PDAC xenograft *in vivo* model. In addition, we performed whole-genome expression analysis of PDAC SP cells, which may guide to CSC-associated characteristics and to potential therapeutic targets. Finally, we explored *in vitro* whether the PDAC SP is enriched in tumourigenic cells as a further characteristic of CSC.

## Methods

### PDAC samples and xenografts

Between 2007 and 2010, PDAC surgical resection specimens were obtained at the University Hospital Leuven (Belgium) from patients after written informed consent (see Table 1). The study was approved by the KU Leuven ethical committee prior to patient recruitment, and received the study number ML3452. Freshly resected tumours were cut into small pieces (2*2 mm) and implanted subcutaneously (s.c.) in the axilla of severe combined immunodeficiency (SCID) mice (male, 6–10 weeks old) to expand tumour material. Tumour growth was evaluated with a caliper on a weekly basis and volume calculated according to the formula: tumour volume = (length x width^2^)/2 [18]. Mice bearing tumours with a minimum volume of 150 mm^3^ were euthanized and tumours were dissected for further analysis. Only first-generation xenograft tumours were used in the experiments described. Hematoxylin-Eosin staining was performed on formalin-fixed sections from original and xenograft tumours.

**Table 1:** Patients’ characteristics with PDAC used for xenografting

**Xenograft no.**	**Sex**	**Age (y)**	**pG**	**pT**	**pN**	**pM**	**pR**	**PNI**	**VI**	**LVI**	**Preop RCT**	**Postop RCT/CT**	**OS (m)**	**DFS (m)**	**SP%**
101*	F	45.5	2	4	1	0	1	1	0	0	0	RCT	35.4	18.5	1.5
110*	M	77.8	2	3	0	0	1	1	1	1	0	0	1.1	1.1	6.0
112*	F	77.8	2	3	1	0	0	1	1	1	0	CT	10.1	3.6	6.8
127	M	52.9	2	2	1	0	0	1	0	0	0	CT	37.7	34.8	6.4
128*	M	53.6	2	3	1	0	1	1	0	0	0	CT	11.2	10.2	6.8
136*	M	78.4	2	3	1	0	0	0	0	0	0	0	37.8	24.5	6.1
151°	M	45.9	2	3	1	0	0	1	1	1	0	CT	13.5	10.3	17.6
169	F	80.5	2	3	1	0	0	0	1	1	0	0	10.2	2.4	4.3
174**^,^°	M	66.9	2	3	0	0	0	1	1	1	0	CT	43.3	19.8	2.7
178**	F	53.2	3	4	1	0	0	1	0	1	0	CT	27.2	27.2	12.0
199	M	52.2	3	3	0	0	1	1	1	1	0	CT	23.5	4.9	5.0
207**^,^°	F	67.0	2	3	0	0	0	0	0	0	0	CT	7.2	5.5	10.0
218	F	62.6	3	3	1	0	0	1	1	1	0	0	0.3	0.3	1.4
223**^,^°	F	57.3	2	2	1	0	0	1	1	1	0	0	24.5	7.4	2.0
229**	F	73.2	3	3	1	0	0	1	1	1	0	0	4.5	4.0	5.4
235**^,^°	F	57.4	1	3	1	0	1	1	0	0	0	CT	19.6	7.7	2.1
241**^,^°	F	74.6	2	3	0	0	0	1	0	0	0	0	6.9	2.9	2.7

* used for microarray analysis; ** used to investigate gemcitabine resistance; ° used for sphere-formation assay.

Abbreviations: F: female; M: male; y: year; p: pathological; G: histopathological grade; T: tumour size; N: lymph node metastasis; M: metastasis; R: resection margin; PNI: perineural invasion; VI: vascular invasion; LVI: lymphovascular invasion; RCT: radiochemotherapy; CT: chemotherapy; OS: overall survival; DFS: disease-free survival; m: month.

### SP analysis

Xenograft tumours (n = 17) were dissociated into single cells using collagenase type IV (1 mg/ml in Medium 199; Invitrogen, Grand Island, NY). Cells were incubated with Hoechst33342 (Sigma-Aldrich, Bornem, Belgium) at a final concentration of 5 μg/ml, and the SP was identified as a side branch of ‘Hoechst low’ cells using dual-wavelength FACS analysis (FACSVantage SE, equipped with FACS DIVA software, version 6.0; BD Biosciences, Erembodegem, Belgium; Hoechst red with 675/20 nm filter and Hoechst blue with 424/44 nm filter). Verapamil (100μM; Sigma-Aldrich) was added to verify the SP phenotype, as it results in the reduction of the side branch by blocking the multidrug transporters. Propidium Iodide (2μg/ml; Sigma-Aldrich) was used to exclude dead cells. For further characterization, tumour cells were immunostained for the endothelial marker CD31 and the hematopoietic marker CD45. After Hoechst incubation, fluorescein (FITC)-labeled anti-mouse or anti-human CD31 and phycoerythrin (PE)-labeled anti-mouse or anti-human CD45 antibodies (BD Biosciences), or PE-labeled anti-human CD133 (Miltenyi Biotec, Bergisch Gladbach, Germany) were added using dilutions according to the manufacturer’s recommendations. Sorted SP and MP cells were established as monolayers and subjected to Cyto-Rich Red staining (BD Biosciences).

### Treatment of mice bearing xenograft tumours with gemcitabine

To investigate resistance of SP cells to gemcitabine, 7 different human PDAC samples were grown in SCID mice (see Table 1). When the tumour reached a volume of approximately 200 mm^3^, one group of mice received gemcitabine (Eli Lilly, Brussels, Belgium; 200 mg/kg body weight intraperitoneally, 1 injection every 3 days, 6 injections in total) and the other group (bearing the corresponding tumours) was injected with vehicle (0.9% NaCl; control group). Tumour diameter was measured every 3 days after the first injection. Three days after the last injection, mice were euthanized and tumours analyzed to determine the proportion of SP cells as described above. Gemcitabine was considered effective when tumour volume decreased at least 50%.

### Whole-genome expression profiling

For RNA extraction, 25000 SP and 25000 MP cells were sorted by FACS into cold lysis solution (RNeasy Micro Kit; Qiagen, Venlo, The Netherlands). RNA was extracted according to the instructions of the manufacturer. RNA quality and concentration were determined using Picochips on a BioAnalyzer 2100 (Agilent Technologies, Santa Clara, CA). Only samples with RNA Integrity Number (RIN) ≥8.0 were used for gene expression profiling by microarray analysis. After Baugh amplification, Cy3 label was incorporated into the cRNA, which was then hybridized onto whole-genome human 44 K oligonucleotide arrays (G4112F, Agilent) [19,20].

Expression values were obtained using the Agilent feature extraction software (version 10.1.1.1) and subjected to quantile normalization. Probes lacking a detection call signal (n = 1666) were omitted from further analysis. The log2-ratios for each SP-MP pair were compared with the Limma (Linear Models for Microarra Data) package of Bioconductor [21]. The contrast SP-MP was tested with a moderate t-statistic (implemented in Limma). The resulting p-values were corrected for multiple testing with Benjamini-Hochberg to control false discovery rate. The Database for Annotation, Visualization and Integrated Discovery (DAVID, version 6.7, http://www.david.abcc.ncifcrf.org) was used to uncover enriched function-related gene groups by gene-annotation enrichment analysis and to reveal enriched KEGG (Kyoto Encyclopedia of Genes and Genomes) pathways. Significance was tested by the EASE score, a modified Fisher’s exact p-value test to adjust for multiple testing. Gene clusters with an enrichment score of >1.5 were retained. To further visualize gene networks, the Search Tool for the Retrieval of Interacting Genes (STRING, http://string.embl.de) was used.

Gene expression data are available from the Gene Expression Omnibus (GEO, http://www.ncbi.nlm.nih.gov/projects/geo/) through series accession number GSE36563.

### Tumourigenic (CSC) activity as analyzed by *in vitro* sphere formation

To investigate sphere-forming capacity, SP and MP subpopulations were sorted by FACS from PDAC xenograft tumours (n = 6; see Table 1), and 40000 cells of each seeded in DMEM/F12 (Invitrogen), supplemented with 0.4% BSA and containing basic fibroblast growth factor (bFGF; 10 ng/ml; R&D Systems, Minneapolis, MN), epidermal growth factor (EGF; 20 ng/ml; R&D Systems), insulin-transferrin-selenium (1:100; Invitrogen) and B27 (1:50; Invitrogen) [22]. The medium was renewed at day 3, and spheres were counted at day 7 to determine the sphere-forming capacity.

### Statistical analysis

Statistical analysis was performed using the Visual data discovery software JMP9 (SAS, Cary, NC). A Wilcoxon Test for nonparametric variables was applied to determine the statistical level of difference between values of PDAC SP and MP and to compare the results from the mouse group treated with gemcitabine and the control group. Statistical significance was determined as p < 0.05, and as p < 0.001 for the microarray analysis.

## Results

### Presence of a SP in human PDAC xenografts

**Figure 1:**
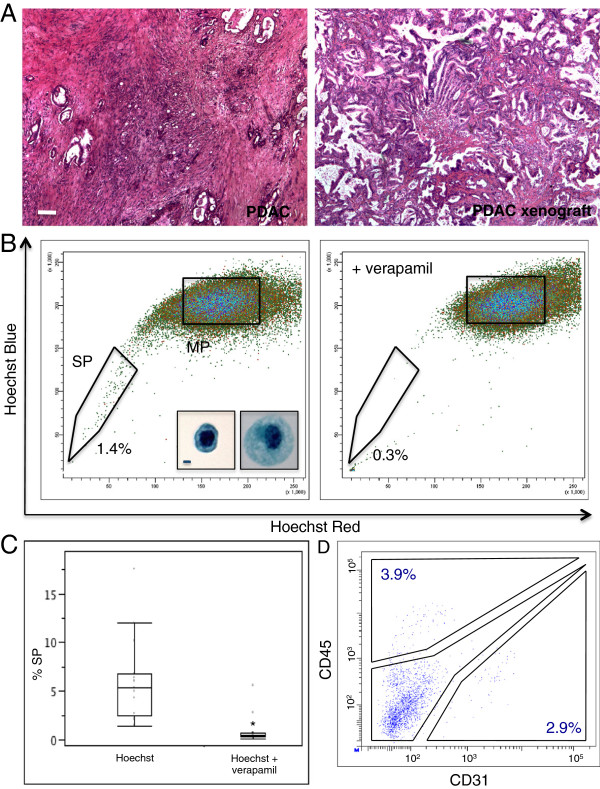
**A SP is present in human PDAC. **(**A**) Hematoxylin-Eosin staining of an original PDAC sample (left) and the corresponding xenograft tumour (right). A representative example is shown. Scale bar = 50 μm. (**B**) SP identification by FACS (left) and control of the SP phenotype with verapamil (right). A representative example with SP proportions is displayed. Inserts show a SP cell (left) and a MP cell (right) after Cyto Rich Red staining. Scale bar = 1 μm. (**C**) Boxplot of the SP proportion, with and without verapamil, of all PDAC xenografts analyzed (n = 17). *, p < 0.05. (**D**) Analysis of CD45 and CD31 expression in the PDAC xenograft SP using FACS. A representative example is shown. Numbers represent the percentage of CD45^+^ cells and of CD31^+^ cells within the SP.

Human PDAC samples (n = 17; Table 1) were expanded by s.c. implantation, each in several SCID mice. All tumours grew in at least one mouse, regardless of the PDAC clinicopathological features or disease stage. Xenograft tumours showed histological resemblance to the original PDAC, although the stromal component was less abundant in the xenograft than in the original tumour (Figure 1A). In all tumours analyzed (n = 17), a SP was identified, ranging from 1.4 to 17.6% of total cells (median: 5.4%; Figure 1B-C; Table 1). Addition of verapamil during incubation with Hoechst decreased the SP proportion to 0.4% (range: 0.1-5.6%), thus confirming the SP phenotype (Figure 1B-C). The SP cells were smaller than the MP cells and had a larger nucleus/cytoplasm ratio, indicative of a poorly differentiated or undifferentiated nature [3] (Figure 1B). No significant correlation was found between the SP size and patients’ survival in this population of 17 PDAC (Table 1). Because it is known that the SP phenotype can co-purify endothelial and hematopoietic cells [20,23], we further analyzed the xenografts for CD31^+^ and CD45^+^ cells. Human epitopes were not detected (data not shown) but mouse CD45^+^ and CD31^+^ cells were found, indicating that host cells were attracted and incorporated into the growing human xenograft tumours. Yet, the proportion of CD45^+^ and CD31^+^ cells in the SP was low (median: 4.1% and 4.5%, respectively; n = 10; Figure 1D).

### The SP is resistant to gemcitabine as assessed *in vivo *

**Figure 2:**
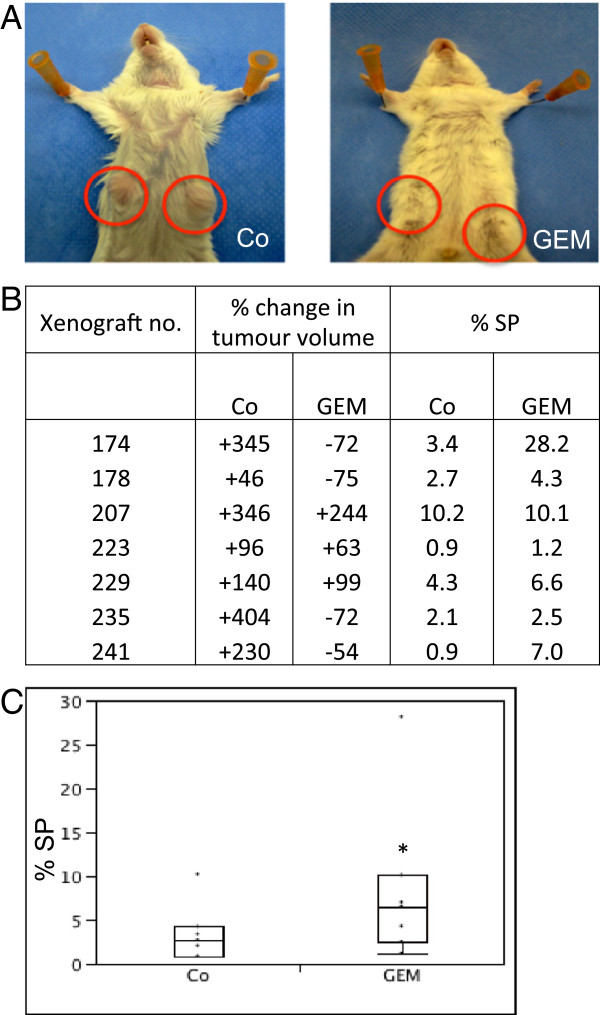
**The SP in human PDAC is resistant to gemcitabine as analyzed*in vivo. ***(**A**) SCID mice with corresponding PDAC xenograft tumours treated with vehicle (control or Co, left) or gemcitabine (GEM, right). Tumours measured 276 mm^3^ and 266 mm^3^ in the control mouse and 144 mm^3^ and 123 mm^3^ in the GEM-treated mouse, respectively. (**B**) Overview of the data, indicating PDAC xenograft number, percent change in tumour volume after treatment with vehicle (Co) or GEM (response to GEM when >50% reduction), and proportion of SP in the tumours after treatment. (**C**) Boxplot of SP proportion in the PDAC xenografts of the vehicle-treated control mice (Co) and the mice treated with GEM (n = 7). *, p < 0.05.

Seven different PDAC samples were grown in SCID mice (see Table 1). Treatment of mice with gemcitabine affected growth of 4 out of 7 xenograft tumours, resulting in an average volume reduction of 72% in comparison with an average tumour expansion in the vehicle-treated control group of 230% (p = 0.030) (Figure 2A-B). After treatment, tumours were excised and analyzed for SP. The tumour SP proportion was larger in mice treated with gemcitabine than in the corresponding controls (median 6.6% *versus* 2.7%, respectively; n = 7; p = 0.028; Figure 2B-C). SP enrichment was even higher when only considering the tumours that responded to gemcitabine with tumour shrinkage (of at least 50%) (median: 4.7% *versus* 1.3%, respectively; n = 4; Figure 2B). CD45^+^ and CD31^+^ cells did not rise in the SP in response to gemcitabine (p = 0.21 and p = 0.66, respectively) when compared to control mice.

### Whole-genome expression analysis of the PDAC xenograft SP reveals upregulation of genes related to therapy resistance

To characterize the SP at the gene expression level, whole-genome expression analysis was performed on SP and MP cells sorted from 5 different xenograft tumours (see Table 1). Microarrays with *human* genome oligonucleotide probes were used, thereby excluding the detection of *mouse* transcripts (such as from the infiltrating CD31^+^ and CD45^+^ SP cells). Comparison revealed that 145 probe sets, representing 121 genes, were differentially expressed between the SP and MP (p < 0.001); 80 genes were upregulated in the SP and 41 genes downregulated (complete list in Additional file 1: Table S1; extract of genes in Table 2, selected on the basis of relevance from the literature and from DAVID analysis as below).

**Table 2:** **Selection of genes differentially expressed in the PDAC xenograft SP*versus *MP**

**Gene Symbol**	**Genbank Accession no.**	**Gene Name**	**Fold SP/MP**	**p-value**
ZAP70	NM_001079	zeta-chain (TCR) associated protein kinase 70 kDa	24.25	0.00002
PRKCQ	NM_006257	protein kinase C, theta	21.56	0.00008
FASLG	NM_000639	Fas ligand (TNF superfamily, member 6)	19.56	0.00003
LEF1	NM_000639	lymphoid enhancer-binding factor 1	16.45	0.00009
PACSIN1	NM_020804	protein kinase C and casein kinase substrate in neurons 1	12.47	0.00015
MEOX2	NM_005924	mesenchyme homeobox 2	8.22	0.00060
STAT4	NM_003151	signal transducer and activator of transcription 4	7.46	0.00050
EMX2	NM_003151	empty spiracles homeobox 2	6.87	0.00006
SOX11	NM_003108	SRY (sex determining region Y)-box 11	6.19	0.00029
FGF7	NM_002009	fibroblast growth factor 7 (keratinocyte growth factor)	5.74	0.00072
FOXG1B	NM_005249	forkhead box G1B	5.62	0.00019
PKNOX2	NM_022062	PBX/knotted 1 homeobox 2	5.58	0.00017
ITGB3	NM_000212	integrin, beta 3 (platelet glycoprotein IIIa, antigen CD61)	5.35	0.00062
KLF12	NM_007249	Kruppel-like factor 12	5.03	0.00048
SNAI2	NM_003068	snail homolog 2 (Drosophila)	4.99	0.00079
TIE1	NM_005424	tyrosine kinase with immunoglobulin-like and EGF-like domains 1	4.76	0.00019
IRX2	AY335940	iroquois homeobox protein 2	4.53	0.00010
EFNB2	NM_004093	ephrin-B2	4.23	0.00016
ETS1	NM_005238	v-ets erythroblastosis virus E26 oncogene homolog 1 (avian)	3.95	0.00095
BCL2L11	NM_138621	BCL2-like 11	3.68	0.00019
GRB10	NM_001001555	growth factor receptor-bound protein 10	3.68	0.00034
NFIB	NM_005596	nuclear factor I/B	3.46	0.00010
INHBB	NM_002193	inhibin, beta B (activin AB beta polypeptide)	3.32	0.00059
TCF7L1	NM_031283	transcription factor 7-like 1 (T-cell specific, HMG-box)	3.14	0.00073
EPC2	NM_053001	enhancer of polycomb homolog 2 (Drosophila)	2.95	0.00090
GATA1	NM_002049	GATA binding protein 1 (globin transcription factor 1)	2.57	0.00082
KITLG	NM_000899	KIT ligand	2.50	0.00052
ERRFI1	NM_018948	ERBB receptor feedback inhibitor 1	2.45	0.00074
CADM1	NM_014333	cell adhesion molecule 1	−22.84	0.00077
TREM2	NM_018965	triggering receptor expressed on myeloid cells 2	−9.90	0.00017
ALOX5AP	NM_001629	arachidonate 5-lipoxygenase-activating protein	−9.32	0.00017
PLA2G7	NM_005084	phospholipase A2, group VII (platelet-activating factor acetylhydrolase, plasma)	−7.66	0.00007
CD74	NM_004355	CD74 molecule, major histocompatibility complex, class II invariant chain	−7.28	0.00006
MADCAM1	NM_130760	mucosal vascular addressin cell adhesion molecule 1	−6.53	0.00004
CD14	NM_130760	CD14 molecule	−5.94	0.00038
CTSA	NM_000308	cathepsin A	−3.29	0.00068
CTSC	NM_001814	cathepsin C	−2.97	0.00085

**Figure 3:**
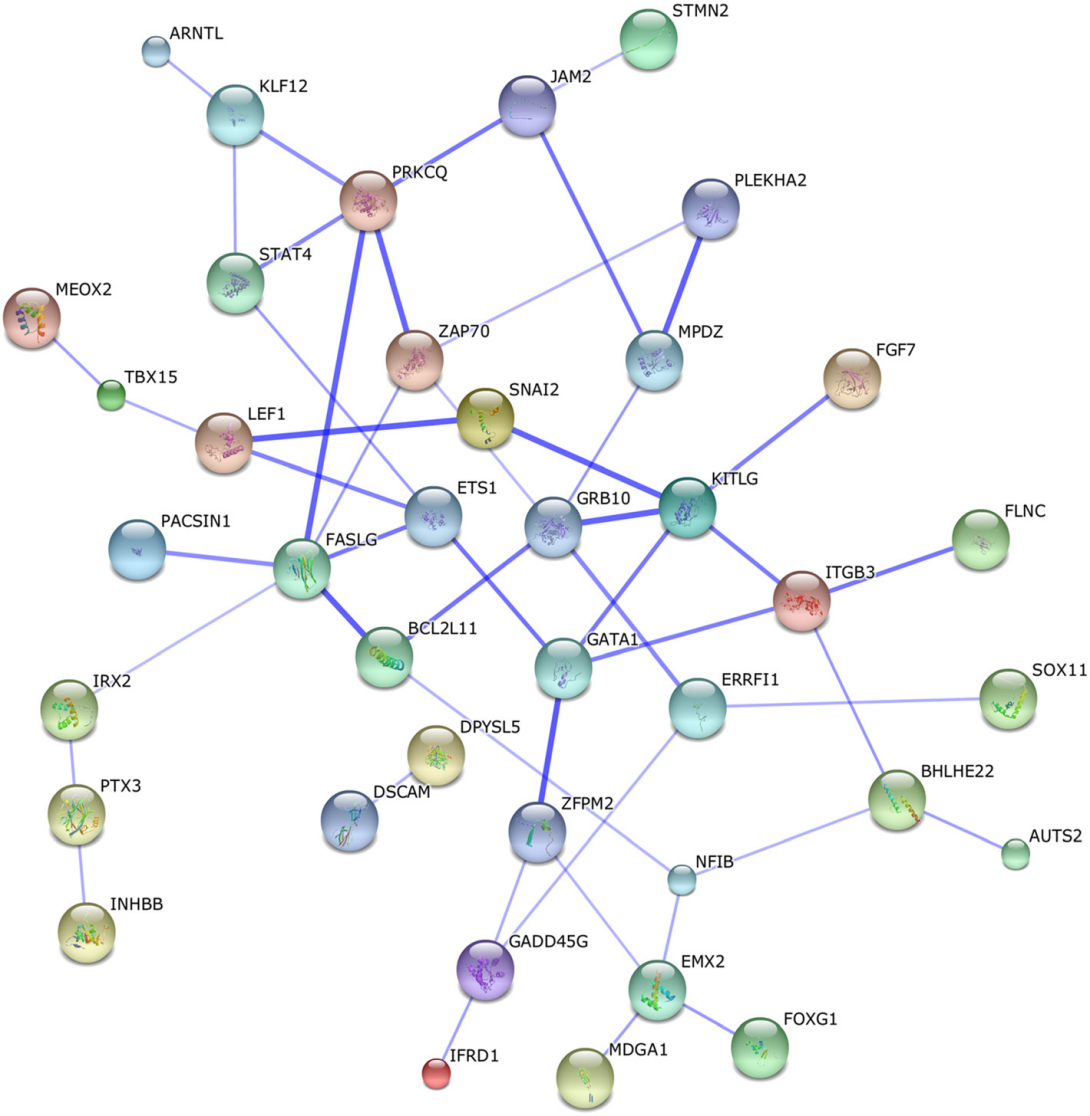
**Interaction network of genes upregulated in the human PDAC SP.** STRING analysis of genes upregulated in the human PDAC xenograft SP *versus* the MP (displayed as ‘evidence view’, i.e. only connected nodes are shown).

Gene-clustering analysis of all differentially expressed genes (p < 0.001) by DAVID showed 3 functionally related groups of genes enriched in the SP: one group of transcription factors, one of adhesion molecules and one of homeobox genes (Table 3). KEGG pathway analysis revealed 3 significantly upregulated pathways in the SP *versus* the MP, including ‘cancer’ and ‘adherens junctions’ (Table 3). Two KEGG pathways were significantly downregulated in the SP, including ‘cell adhesion molecules’. Visualization of the interaction network of SP-upregulated genes by STRING analysis (Figure 3) reveals that genes involved in chemoresistance [*ETS1*, KIT ligand ( *KITLG*) or stem cell factor ( *SCF*), *SNAI2*], regulation of apoptosis ( *FASLG, GRB10, BCL2L11, ETS1, SNAI2*), epithelial-mesenchymal transition (EMT) ( *SNAI2, LEF1*) and tumourigenesis (oncogenes like *FGF7, GATA1, KITLG, ETS1*) occupy a central position. Moreover, multidrug transporters, linked to chemoresistance and some considered responsible for the SP phenotype, also show a clear tendency of upregulation in the SP ( *ABCG2*, 3.63 fold, p = 0.006; *ABCA9*, 3.66-fold, p = 0.003).

**Table 3:** Gene-function analysis of all differentially expressed genes between PDAC xenograft SP and MP using DAVID

**A. ENRICHED GENE CLUSTERS**		
Gene group 1**: Transcription Factors** Enrichment Score: 1.78		
1	TCF7L1	transcription factor 7-like 1 (T-cell specific, HMG-box)
2	SNAI2	snail homolog 2 (Drosophila)
3	CNOT4	CCR4-NOT transcription complex, subunit 4
4	ZBTB10	zinc finger and BTB domain containing 10
5	KLF12	Kruppel-like factor 12
6	ZFPM2	zinc finger protein, multitype 2
7	STAT4	signal transducer and activator of transcription 4
8	OSR2	odd-skipped related 2 (Drosophila)
9	IKZF2	IKAROS family zinc finger 2 (Helios)
10	PHF6	PHD finger protein 6
11	ZFHX4	zinc finger homeobox 4
12	SOX11	SRY (sex determining region Y)-box 11
13	CITED4	Cbp/p300-interacting transactivator, with Glu/Asp-rich carboxy-terminal domain, 4
14	ARNTL	aryl hydrocarbon receptor nuclear translocator-like
15	NFIB	nuclear factor I/B
16	GATA1	GATA binding protein 1 (globin transcription factor 1)
17	ETS	v-ets erythroblastosis virus E26 oncogene homolog 1 (avian)
18	LEF1	lymphoid enhancer-binding factor 1
Gene group 2: **Adhesion Molecules** Enrichment Score: 1.71		
1	HLA-DPB1	major histocompatibility complex, class II, DP beta 1
2	MADCAM1	mucosal vascular addressin cell adhesion molecule 1
3	TREM2	triggering receptor expressed on myeloid cells 2
4	EFNB2	ephrin-B2
5	IGSF6	immunoglobulin superfamily, member 6
6	LRFN5	leucine rich repeat and fibronectin type III domain containing 5
7	DSCAM	Down syndrome cell adhesion molecule
8	JAM2	junctional adhesion molecule 2
9	MDGA1	MAM domain containing glycosylphosphatidylinositol anchor 1
Gene group 3: **Homeobox Genes** Enrichment Score: 1.51		
1	PKNOX2	PBX/knotted 1 homeobox 2
2	MEOX2	mesenchyme homeobox 2
3	SPIC	Spi-C transcription factor (Spi-1/PU.1 related)
4	IRX2	iroquois homeobox 2
5	EMX2	empty spiracles homeobox 2
**B. KEGG PATHWAYS**		
**Upregulated in SP**		
	Number of genes	genes
Arrhythmogenic rightventricular cardiomyopathy	4	GJA1, ITGB3, LEF1, TCFL1
Pathways in cancer	6	TCF7L1, KITL, FASL, LEF1, FGF7, ETS1
Adherens junction	3	LEF1, SNAI2, TCF7L1
**Downregulated in SP**		
	Number of genes	genes
Lysosome	3	CTSA, CTSC, SLC11A1
Cell adhesion molecules (CAMs)	3	CADM1, HLA-DPB1, MADCAM1

### The SP is enriched in sphere-forming cells

**Figure 4:**
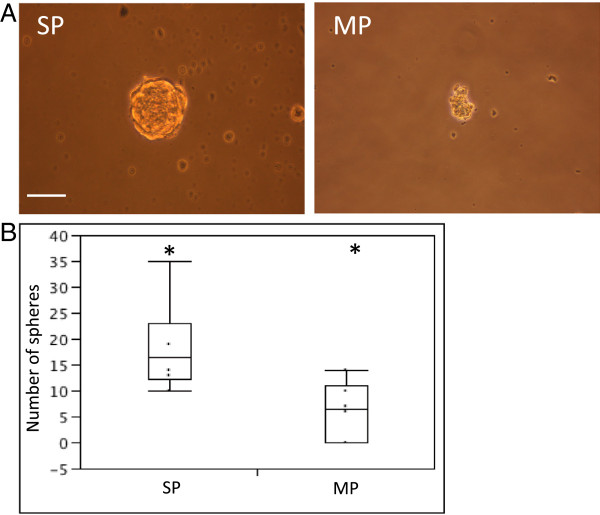
**The human PDAC SP displays higher sphere-forming capacity than the MP.** (**A**) Spheres grown from CD45^-^/CD31^-^ SP cells (left) and CD45^-^/CD31^-^ MP cells (right) sorted from human PDAC xenografts. Representative examples are shown. Scale bar = 50 μm. (**B**) Boxplot of the number of spheres obtained (n = 6). *, p < 0.05.

Tumourigenic (CSC) activity was analyzed *in vitro* using the sphere-forming assay [22]. SP and MP from xenograft tumours were first depleted from the (murine) endothelial and immune cells by FACS and then seeded in defined culture conditions (see Methods). Viability of the sorted SP and MP cells was identical (data not shown). The CD45^-^/CD31^-^ SP generated spheres in all experiments (n = 6; median number of spheres: 16; range: 10–35; Figure 4). In contrast, the CD45^-^/CD31^-^ MP did not consistently generate spheres (not in 2 out of the 6 experiments) and the spheres obtained were lower in number (range: 0–15; median: 8; p = 0.016 *versus* SP), less well-formed, and smaller in size (Figure 4). These findings indicate that SP cells have a higher sphere-forming capacity than MP cells. It was not possible to assess the propagation (self-renewal) capacity of the sphere-forming cells because the number of spheres obtained was too low and the dispersion did not yield enough (viable) cells.

## Discussion

To date, SP analysis in pancreatic cancer has been limited to cultured cell lines [6-8,24,25]. In the present study, we demonstrate for the first time the presence of a SP in human PDAC samples using first-generation xenografts, histologically resemblant to the original tumour samples except for the stromal component. We showed, using a xenograft *in vivo* model, that the SP is more resistant to gemcitabine than the other tumour cells, and therefore may represent a potential therapeutic target. The response rate of the xenograft tumours to gemcitabine (4 out of 7) was higher than the known response rate in human patients (lower than 20%), which may be due to the reduced stromal component in the xenografts, thus improving drug delivery to the tumour cells (see [3,4]).

Whole-genome expression profiling of the SP demonstrated the expression of genes involved in cancer pathways, particularly chemoresistance and EMT. In many cancers, the proto-oncogene *ETS1* plays a role in chemoresistance and invasion [26]. In PDAC cell lines, *ETS1* expression has been linked to gemcitabine resistance and to invasiveness by induction of matrix metalloproteinase (MMP) 2 [27]. We also found upregulation of *MMP2* in the PDAC SP, although at the border of statistical significance (3.12 fold; p = 0.003). Equally, *KITLG* ( *SCF*) increases invasive capacity in PDAC cell lines, as well as cell proliferation [28,29]. The zinc finger transcription factor *SNAI2* ( *SLUG*) is involved in chemo- and radioresistance (e.g. through anti-apoptotic mechanisms) [30]. Moreover, SNAI2 is a core regulator of EMT, a key process in cancer pathogenesis and tumour progression by which epithelial cells acquire a mesenchymal phenotype with invasive and migratory properties. EMT is considered to play an important role in tumour resistance and metastasis. The SP of the pancreatic cell line PANC-1 has been shown to possess superior potential for EMT when compared to the MP [24]. Recently, SNAI2 was shown to promote EMT, invasion and metastasis in a pancreatic cancer cell line [31,32]. EMT involves the downregulation of cell adhesion molecules like E-cadherin. In the present study, *E-cadherin* was significantly downregulated in 3 of the 5 PDAC samples analyzed (data not shown). SNAI2 can act as a transcriptional repressor of E-cadherin. However, in pancreatic cancer cell lines, SNAI2 was found only a weak suppressor of E-cadherin [32], whereas in human PDAC, no significant correlation could be observed between SNAI2 and E-cadherin [32,33]. LEF1 (lymphoid enhancer factor 1), a nuclear transducer of the Wnt signaling pathway, also plays a key role in EMT. In addition, *LEF1* expression in human pancreatic cancer correlates with advanced tumour stages [34]. TCF7L1 is known to form a complex with LEF1 to achieve DNA-binding ability. Finally, other signaling proteins of the Wnt pathway (*Wnt7b, DVL1, FZD1, FZD4, FZD5*) as well as components of the TGFβ/BMP pathway ( *TGFβ1, BMP1, BMPP2Ra, SMURF2*) were upregulated in the SP *versus* MP, although not reaching statistical significance (0.001 < p < 0.05; data not shown).

Protection against apoptosis represents a further mechanism of therapy resistance. In our current study, *BCL2L11* and *FASLG* are upregulated in the SP. In studies with pancreatic cancer cell lines, BCL2L11 has been correlated with apoptotic resistance as well as with metastatic potential [35,36]. FASLG may regulate immune evasion of tumour cells by inducing apoptosis in cytotoxic T lymphocytes. FASLG has been reported to play a role in the aggressiveness of PDAC, potentially through this immune escape mechanism [37].

Finally, the multidrug transporter *ABCG2* is highly expressed in the SP, suggesting a role in chemoresistance and at the same time supporting the SP phenotype which is, at least partially, linked to activity of this pump. In pancreatic cancer cell lines, *ABCG2* was also found to be upregulated in the SP [6,8] and associated with chemotherapy resistance [38]. In addition, *ABCA9*, another membrane transporter linked to chemoresistance in some cancers like malignant melanoma [39], was found upregulated in the PDAC SP in our study.

In multiple types of cancer, the SP displays properties reminiscent of CSC. In the present study, we show that the SP of PDAC is enriched in cells that generate spheres. Although sphere formation is regarded as an *in-vitro* assay for CSC (tumourigenic) activity (see e.g. [22]), it should be noted that this link is not always present as reported, for instance, in high-grade glioma [40]. In order to demonstrate tumourigenic (CSC) activity of the PDAC SP, further study is needed that analyzes *in-vivo* tumour growth after implantation of the (purified) population in immunodeficient mice. Given the essential interactions of cancer cells with their microenvironment for tumour development, transplantation within the pancreas would be most appropriate but technically highly demanding. Yet, some expression characteristics may already suggest a CSC(−like) phenotype including expression of genes associated with chemoresistance (see above), with the Wnt pathway and with EMT. Indeed, the latter process has recently been uncovered as a key promoter of the generation and activity of CSC [41]. Moreover, KITLG (SCF) has been linked to CSC in prostate [42] and lung cancer [43]. In the present study, expression of the previously proposed CSC membrane markers in pancreatic cancer (i.e. CD24^+^CD44^+^ESA^+^[16,44] and CD133^+^[17]) was not found upregulated in the PDAC xenograft SP, neither in microarray analysis, nor in flow-cytometric examination (1.0% CD133^+^ cells in the SP and 0.7% in the MP; n = 2) (data not shown). Noteworthy, expression of the two previously defined sets of markers (CD24^+^CD44^+^ESA^+^ and CD133^+^) did also not completely, or only minimally, overlap (10-40%) [16]. Thus, CSC in pancreatic cancer, and the link with the SP, need further investigation.

## Conclusions

Our study revealed the presence of a SP in human PDAC, displaying chemoresistance and CSC-associated activity, as well as expression of genes involved in both processes. SP cells thus may represent interesting targets for new and more efficient therapeutic strategies. Chemoresistance, anti-apoptosis and EMT genes identified may guide us to potential molecular targets.

## Abbreviations

PDAC: Pancreatic ductal adenocarcinoma; SP: Side population; MP: Main population; FACS: Fluorescence-activated cell sorter; CSC: Cancer stem cell(s); s.c.: Subcutaneously; SCID: Severe combined immunodeficiency; DAVID: Database for annotation: Visualization and Integrated Discovery; KEGG: Kyoto Encyclopedia of Genes and Genomes; STRING: Search Tool for the Retrieval of Interacting Genes/Proteins; EMT: Epithelial-mesenchymal transition.

## Competing interests

The authors declare that they have no competing interests

## Authors’ contributions

AVDB designed and performed the study, analyzed the data and wrote the manuscript. LG trained AVDB in the techniques used and helped in data collection. BT participated in study design and analysis and in writing. HV participated in the design and analysis of the study and wrote the manuscript. All authors read and approved the final manuscript.

## Pre-publication history

The pre-publication history for this paper can be accessed here:

http://www.biomedcentral.com/1471-2407/12/354/prepub

## Supplementary Material

Additional file 1: Table S1 Complete list of genes differentially expressed in the PDAC SP versus MP (p < 0.001). Click here for file
